# Influence of dimethyl dicarbonate on the resistance of *Escherichia coli* to a combined UV-Heat treatment in apple juice

**DOI:** 10.3389/fmicb.2015.00501

**Published:** 2015-05-19

**Authors:** Maria Gouma, Elisa Gayán, Javier Raso, Santiago Condón, Ignacio Álvarez

**Affiliations:** ^1^Departamento de Producción Animal y Ciencia de los Alimentos, Tecnología de los Alimentos, Facultad de Veterinaria, Universidad de ZaragozaZaragoza, Spain; ^2^Laboratory of Food Microbiology, Department of Microbial and Molecular Systems, Katholieke Universiteit LeuvenLeuven Belgium

**Keywords:** *Escherichia coli*, UV-C light, heat, apple juice, dimethyl dicarbonate

## Abstract

Commercial apple juice inoculated with *Escherichia coli* was treated with UV-C, heat (55°C) and dimethyl dicarbonate – DMDC (25, 50, and 75 mg/L)-, applied separately and in combination, in order to investigate the possibility of synergistic lethal effects. The inactivation levels resulting from each treatment applied individually for a maximum treatment time of 3.58 min were limited, reaching 1.2, 2.9, and 0.06 log_10_ reductions for UV, heat, and DMDC (75 mg/L), respectively. However, all the investigated combinations resulted in a synergistic lethal effect, reducing the total treatment time and UV dose, with the synergistic lethal effect being higher when larger concentrations of DMDC were added to the apple juice. The addition of 75 mg/L of DMDC prior to the combined UV-C light treatment at 55°C resulted in 5 log_10_ reductions after only 1.8 min, reducing the treatment time and UV dose of the combined UV-Heat treatment by 44%.

## Introduction

Apple juice is one of the most popular juices in developed countries due to its pleasant organoleptic properties, ranking third in the EU fruit juice and nectars market (AIJN, 2012). Several foodborne disease outbreaks associated with the consumption of apple juice have implicated *Escherichia coli* O157:H7 as a potential pathogen (Besser et al., 1993; Cody et al., 1999; Vojdani et al., 2008). In response, the (United States Food and Drug Administration [USFDA], 2001) considered this microorganism a pertinent pathogen for juices, requiring that juice processors apply a treatment that results in at least 5-log_10_ reductions of its population in fruit and vegetable juices.

Thermal pasteurization has been predominantly used in the juice industry to avoid this danger. However, its adverse effects on the nutritional and sensorial quality properties of the end product—as well as consumers’ demands for minimally processed, fresh-like products—have impelled food research to develop non-thermal food processing technologies. Among them, UV-C radiation has shown a considerable potential effect for juice pasteurization. In fact, the (National Advisory Committee on Microbiological Criteria for Foods of the United States Department of Agriculture [NACMCF], 2006) revised the definition of “pasteurization” in 2004 and included UV irradiation as an alternative to heat that can be used for pasteurization purposes. UV-C radiation is considered a promising alternative to traditional heat treatments due to its various advantages such as the effectiveness in the inactivation of a wide range of both pathogen and spoilage microorganisms, the absence of toxic residues, the simple operation and maintenance and the low energy consumption (Guerrero-Beltrán and Barbosa-Cánovas, 2004). Nevertheless, its high dependence on the optical properties of the treated liquid (e.g., turbidity, absorption coefficient) renders it insufficient to achieve the required 5-log_10_ reductions imposed by the USFDA under industrially applicable treatment conditions (Murakami et al., 2006; Koutchma, 2008). To overcome this disadvantage, research has focused on the development of combined processes based on the hurdle technology approach, aiming to increase the lethal effect of UV-C radiation. To this direction, combinations with other non-thermal technologies and mild heat treatments as well as its combination with antimicrobial agents have been investigated (Koivunen and Heinonen-Tanski, 2005; Wu et al., 2011). For instance, UV-C treatments in combination with pulsed electric fields (PEFs; Gachovska et al., 2008) and high-intensity ultrasounds (US; Char et al., 2010) have resulted in an additive lethal effect. On the contrary, the combination of UV-C radiation and mild heat (UV-H) has demonstrated a synergistic inactivation of *E. coli* in fruit juices (Gayán et al., 2012a,b). In particular, a combined UV-H treatment (3.9 J/mL at 55°C for 3.6 min) has been designed, with which it has been possible to inactivate 5-log_10_ cycles of a cocktail of pathogenic *E. coli* strains in apple juice without impairing the quality attributes of the product (Gayán et al., 2012a). However, this treatment could be too long to be applied on an industrial scale. Therefore, new combined treatments that enable reducing the intensity of these treatments could be of interest.

Dimethyl dicarbonate is a powerful microbial inhibitor by inactivating cellular enzymes (Bartowsky, 2009). It is primarily used to prevent yeast spoilage in wine, where it is added before bottling, and undergoes complete hydrolysis within a few hours to methanol and carbon dioxide, which are both natural constituents of the product. Its effectiveness against yeasts in alcoholic beverages has been well proven and evaluated (Daudt and Ough, 1980; Costa et al., 2008). However, its lethal effectiveness varies among species and strains (Bartowsky, 2009). Studies on grape must have demonstrated that bacteria were more resistant than yeast to DMDC (Delfini et al., 2002). DMDC can also be used as a microbial control agent in non-carbonated 100% juice beverages up to a maximum concentration of 250 ppm (United States Food and Drug Administration [USFDA], 2000). In this case, beverages must be produced under good manufacturing conditions and their microbial load must first be reduced by current technologies such as heat treatment or filtration prior to the addition of DMDC (United States Food and Drug Administration [USFDA], 2013). To our knowledge, there is a dearth of published data on the use of DMDC in the inactivation of pathogenic bacteria in fruit juices and, in particular, on its combined application with mild heat treatments and UV-H light. Thus, the objective of this study was to investigate the inactivation effect of UV-C light, mild heat and DMDC alone and in combination against *E. coli* in clarified apple juice.

## Materials and Methods

### Bacterial Culture

*Escherichia coli* strain STCC 4201, provided by the Spanish Type Culture Collection (STCC), was used. The bacterial culture was frozen at -80°C in cryovials. Stationary-phase cultures were prepared by inoculating 5 mL of tryptone soy broth (Oxoid, Hampshire, UK) supplemented with 0.6% (w/v) yeast extract (Oxoid; TSBYE) with a colony grown on tryptone soy agar (Oxoid) supplemented with 0.6% (w/v) yeast extract (TSAYE). The precultures were incubated at 35°C for 6 h in a shaking incubator. Fifty microliters of the precultures were inoculated into 50 mL of fresh TSBYE and incubated for 24 h under the same conditions, which resulted in stationary-phase cultures containing approximately 2 × 10^9^ CFU/mL.

### Treatment Medium

Commercial clarified apple juice (Pascual S.L., Córdoba, Spain) was purchased from a local market. It presented a pH of 3.6, an absorption coefficient of 24.9 cm^-1^ and a turbidity of 2.4 NTU. Turbidity was measured using an HI 83749 nephelometer (Hanna Instrument, Szeged, Hungary). The pH measurements (20 ± 1°C) were performed with a pH meter (Basic 20 pH meter; Crison Instrument, Barcelona, Spain) with a glass electrode (Crison) and calibrated before measurement at pH 4.0, 7.0, and 9.0. Absorbance of apple juice was measured at 254 nm using a Libra S12 spectrophotometer (Biochrom Ltd., Cambridge, UK). Sample solutions were diluted and evaluated using quartz cuvettes (Hellma, Móllheim, Germany) with path lengths of 1, 2, and 10 mm. The absorption coefficient of the sample solution was determined by the slope of the absorbance versus path length, correcting the dilution factor.

When DMDC was used, a stock solution of DMDC (≥99%; Merck, Darmstad, Germany) was purchased, and after being divided into smaller volumes it was stored at -80°C. The solution was brought to room temperature before its use. To evaluate the lethality of DMDC, different concentrations (from 0 to 250 ppm) of the compound were added to 10 mL of apple juice previously inoculated with 10^6^ CFU/mL of *E. coli* population. At preset treatment times, 0.1 mL samples were taken, immediately diluted in 0.1% peptone water (Oxoid) and then poured into plates using TSAYE.

### UV-C, Heat, and UV-H Treatments

All the treatments performed in the study were carried out with the equipment previously described by Gayán et al. (2011). The system consisted of eight individual annular thin-film flow-through reactors connected sequentially. Each reactor consisted of a low-pressure UV lamp fixed at the axis of an outer glass tube and enclosed by a quartz tube. In the annular gap (2 mm) a stainless steel coil spring was introduced in order to improve the flow’s turbulence. To conduct UV-H treatments, the entire unit was submerged in a 90-L water tank heated by the circulating water of a peripheral thermostatic bath. A heating/cooling coil exchanger was placed before the inlet of the first reactor and submerged in the water bath to ensure that the treatment medium reached the desired temperature before entering the reactor. Heat treatments were performed using the same equipment with the lamps switched off.

The apple juice was inoculated with the bacterial suspension to achieve approximately 10^6^ CFU/mL. After the addition of the DMDC solution (0, 25, 50, or 75 mg/L), the juice was thoroughly mixed and immediately pumped through the equipment at a flow rate of 8.5 L/h. When the flow rate stabilized, samples were withdrawn through the sampling valves at the outlet of each reactor. Then, appropriate dilutions of samples were done and immediately pour-plated.

### Incubation of Treated Samples and Survival Counting

Tryptone soy agar with yeast extract (TSAYE; Oxoid) was used as the recovery medium, and plates were incubated for 24 h at 35°C. Previous experiments demonstrated that longer incubation times did not change the number of survivors. After incubation, colony forming units (CFU) were counted with an improved image analyzer automatic colony counter (Protos, Synoptics, Cambridge, UK), as described by Condón et al. (1996). A detection limit of 30 CFU/plate was set. Each experiment was performed at least three times on separate days. Average results are presented in tables and figures.

## Results and Discussion

It has been demonstrated that the application of UV-C light at moderate temperatures has resulted in a synergistic microbial lethal effect when liquid food products are treated (Geveke, 2008; Gayán et al., 2012a,b). More specifically, in the case of juices, a temperature of 55°C and a treatment time of 3.6 min and a UV dose of 3.9 J/mL have been indicated as the optimum conditions to exploit the synergistic effect resulting from the combined UV-H treatment, reducing five or more log_10_ cycles of different pathogenic microorganisms, including *E. coli* and *Listeria monocytogenes* (Gayán et al., 2012a,b, 2015). In spite of these promising results, the required processing times to achieve the 5-log_10_ reductions demanded by the FDA for the pasteurization of fruit juices should be too long for the industrial application of the technology. Therefore, the purpose of this investigation was to study the occurrence of the synergistic effect of the proposed UV-H process when antimicrobials like DMDC are added to the juice. The existence of this synergism could reduce the treatment time or the UV dose to reach the required 5-Log_10_ reductions, in this case, of *E. coli*. Thus, apple juice inoculated with *E. coli* STCC 4201 was treated with UV-C light, mild heat (55°C) and different concentrations of DMDC (25, 50, and 75 mg/L), applied separately and in combination. *E. coli* STCC 4201 was chosen because it has been proved to be the most UV resistant among different *E. coli* strains (Gayán et al., 2011).

**FIGURE 1:**
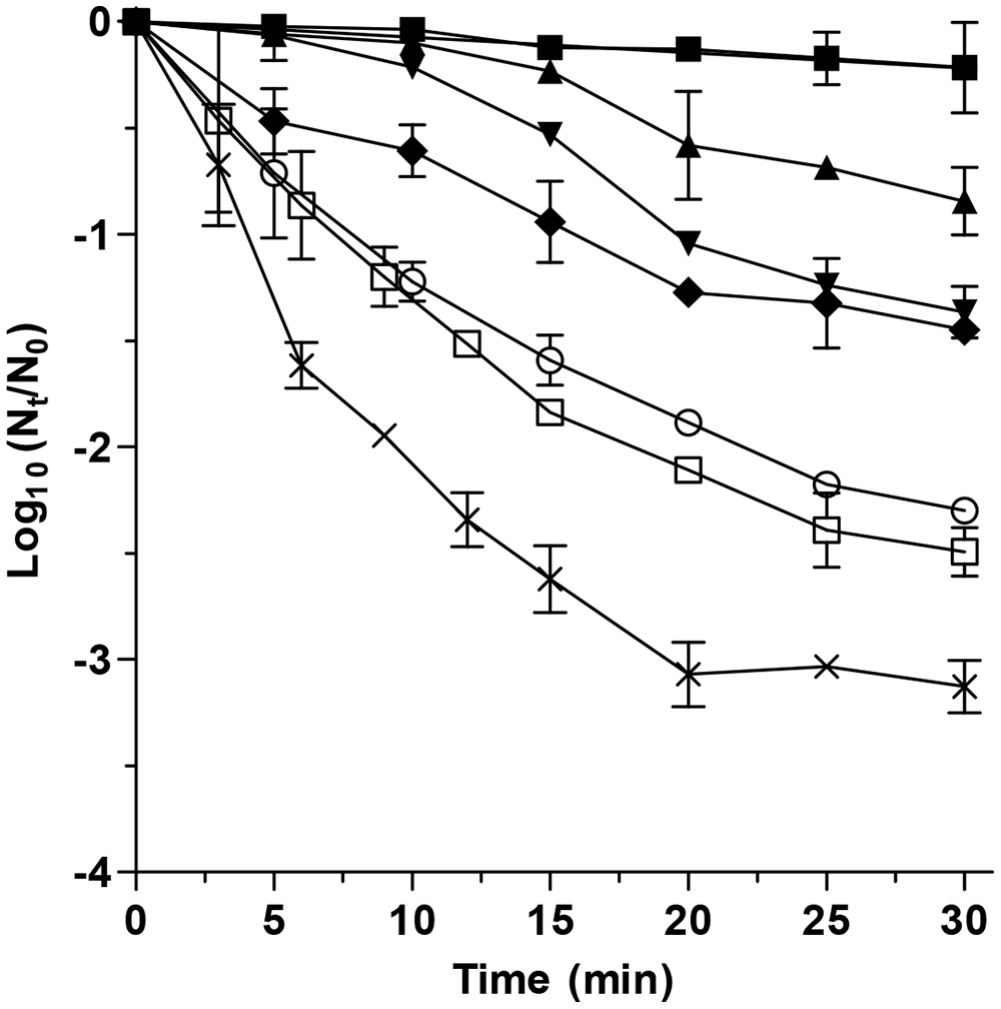
**Inactivation of *Escherichia coli* STCC 4201 after different exposition times to 0 ppm (●), 25 ppm (■), 50 ppm (▲), 75 ppm (▼), 100 ppm (♦), 150 ppm (❍), 200 ppm (☐), and 250 ppm (⨯) of dimethyl dicarbonate (DMDC) at room temperature in clarified apple juice**.

In a first approach and in order to evaluate the lethality of DMDC, *E. coli* inactivation over time due to its exposition to different concentrations of this compound added to clarified apple juice was investigated at 25°C (**Figure 1**). As observed, microbial inactivation increased with time and concentration, enabling a reduction of up to 3-log_10_ cycles after 30 min and 250 ppm, which is the maximum concentration that the FDA permits in non-carbonated 100% juice beverages (United States Food and Drug Administration [USFDA], 2000). Published data on the inactivation of *E. coli* by DMDC in liquid food vary strongly. 1.66 log_10_ cycles reduction of *E. coli* ATCC 8739 in litchi juice was achieved as exposed to 150 mg/L DMDC at 30°C for 1 h, and the inactivation rate during initial 1 h was far greater than during the remaining 5 h (Yu et al., 2014). Fisher and Golden (1998) observed that *E. coli* O157:H7 (initial concentration 10^7^ CFU/mL) survived for up to 3, 9, and 2 days in apple cider containing 250 ppm of DMDC at 4, 10, and 25°C, respectively. A 5-log_10_ reduction of *E. coli* O157:H7 in apple cider was achieved with 250 ppm of DMDC with a holding time of 1.30 h at room temperature (Basaran-Akgul et al., 2009). In this investigation and in order to find out the synergistic lethal effects when combined with UV and heat, the maximum concentration of DMDC that did not affect the number of *E. coli* survivors during a maximum processing time of 10 min was used. Indeed, the longest processing time for the UV and heat process was of 3.6 min. Therefore, concentrations of 75 ppm did not interfere with *E. coli* inactivation, and thus, this concentration was added to apple juice for the following investigations.

**Table 1** summarizes the inactivation of *E. coli* STCC 4201 achieved in apple juice after the application of the maximum UV-H treatment (3.9 J/mL and 3.58 min), which was possible to apply in our facility at a temperature of 55°C. For comparisons, the lethality of the different individual treatments in presence or not of distinct concentrations of DMDC has been included. When the achieved lethality was higher than that of the detection limit of the recovery technique, treatment time was reduced. As observed, the inactivation levels resulting from each treatment applied individually for the maximum applied treatment time were limited, with the heat treatment being the most effective, reaching approximately 3-log_10_ cycles of inactivation. The addition of DMDC synergistically increased the lethality of both heat and UV radiation, mainly when the DMDC concentration was higher than 25 mg/L. At this concentration, the lethal effect was additive: over 25 mg/L, the higher the concentration the greater the synergistic lethal effect. Thus, the addition of 75 mg/L of DMDC (0.06 log_10_ reductions when DMDC was used alone) increased by 1.5 log_10_ cycles the inactivation effect of heat (2.9 log_10_ reductions when heat was used alone), diminishing the *E. coli* population by 4.4 log_10_ cycles after 3.58 min of treatment. When 50 mg/L of DMDC was added, the lethality increased by 0.9 log_10_ cycles. A similar synergistic lethal effect was observed when 75 or 50 mg/L of DMDC were added before the UV treatment. The final inactivation levels reached with the combined processes were 2.9 and 2.3 log_10_ reductions when 75 or 50 mg/L of DMDC were added, respectively, which corresponded to an increase of 1.7 and 1.1 log_10_ cycles compared to the addition of the lethalities of the individual treatments, respectively. Similar results have been described in literature on the combined effect of DMDC with UV light. Thus, it has been demonstrated that the concurrent use of UV (18.3 mJ/cm^2^) and DMDC (250 and 500 ppm) on the inactivation of *L. monocytogenes* in chilled brine was more effective than either of the treatments alone (Parikh et al., 2011). Halim et al. (2012) reported that the addition of DMDC (15 μL/100 mL) to red pitaya juice prior to UV treatment resulted in a twofold microbial reduction compared to individual treatments. Quicho (2005) reported that DMDC (75 and 150 ppm) was effective in reducing *E. coli* ATCC 25922 population in apple cider in conjunction with UV (13.7 mJ/mL), producing more than 6 log_10_ reductions when added prior to UV treatment, when the latter alone produced a inactivation of 4.7 log_10_ cycles. The higher level of inactivation obtained in that investigation could be due to a less resistant strain of *E. coli* was used. Daudt and Ough (1980) reported that the amount of DMDC to sterilize wine inoculated with various strains of yeasts depended mainly on the yeast strain. Moreover, the applied UV dose was manually recorded using sensors that were located on the outermost wall of the interior of the flow chamber, which does not correspond to the UV dose received by the cells in the medium. In addition, no information was indicated concerning the optical properties of the apple cider such as the absorption coefficient and the turbidity, that play a key role in the inactivation efficacy of UV treatment (Koutchma, 2008). The presence of alcohol in apple cider can also result in an increased effectiveness of DMDC (Porter and Ough, 1982). On the other hand, various studies have focused on the effect of UV light on *E. coli* populations in apple juice (Wright et al., 2000; Basaran et al., 2004; Franz et al., 2009; Müller et al., 2011). Nevertheless, a comparison of the data obtained in this investigation with other published data is difficult to do based on UV dosage only due to substantial differences in the conformation and geometry of the UV equipment used in each case, flow pattern and optical properties of the treatment medium (turbidity and absorption coefficient), which play an important role in UV germicidal efficacy (Koutchma et al., 2004).

**Table 1:** Log_10_ cycles of inactivation of *Escherichia coli* STCC 4201 in apple juice after different heat, UV, and UV-H treatments in presence of different concentrations of dimethyl dicarbonate (DMDC).

Treatment	Log_10_ cycles of inactivation	Treatment time (min)
UV	1.2 (0.1)	3.58
HEAT (55°C)	2.9 (0.5)	3.58
DMDC (75 mg/L)	0.06 (0.01)	3.58
HEAT + 25 mg/L	3.2 (0.6)	3.58
HEAT + 50 mg/L	3.8 (0.2)	3.58
HEAT + 75 mg/L	4.4 (0.1)	3.58
UV + HEAT	5.1 (0.1)	3.20
UV + 25 mg/L	1.63 (0.02)	3.58
UV + 50 mg/L	2.3 (0.2)	3.58
UV + 75 mg/L	2.9 (0.1)	3.58
UV + HEAT + 25 mg/L	>6.0	3.58
UV + HEAT + 50 mg/L	>6.0	2.67
UV + HEAT + 75 mg/L	>6.0	2.23

*Numbers in parentheses indicate the standard deviations.*

From **Table 1**, it can also be observed that the maximum lethal effect obtained in this investigation was achieved when the three hurdles were combined simultaneously. Thus, the treatment of a clarified apple juice with UV and mild heat (55°C) ensured more than 6 log_10_ cycles of inactivation in 3.58, 2.67, and 2.23 min of treatment time when 25, 50, or 75 mg/L of DMDC were added, respectively. These results indicate a synergistic lethal effect of at least 1.8 log_10_ reductions when only 25 mg/L of DMDC was used after applying the UV-H combined process. With higher concentrations of DMDC, the synergistic lethal effect would be higher since the treatment time to achieve more than 6 log_10_ reductions (maximum plate count detection limit in this investigation) was reduced.

**FIGURE 2:**
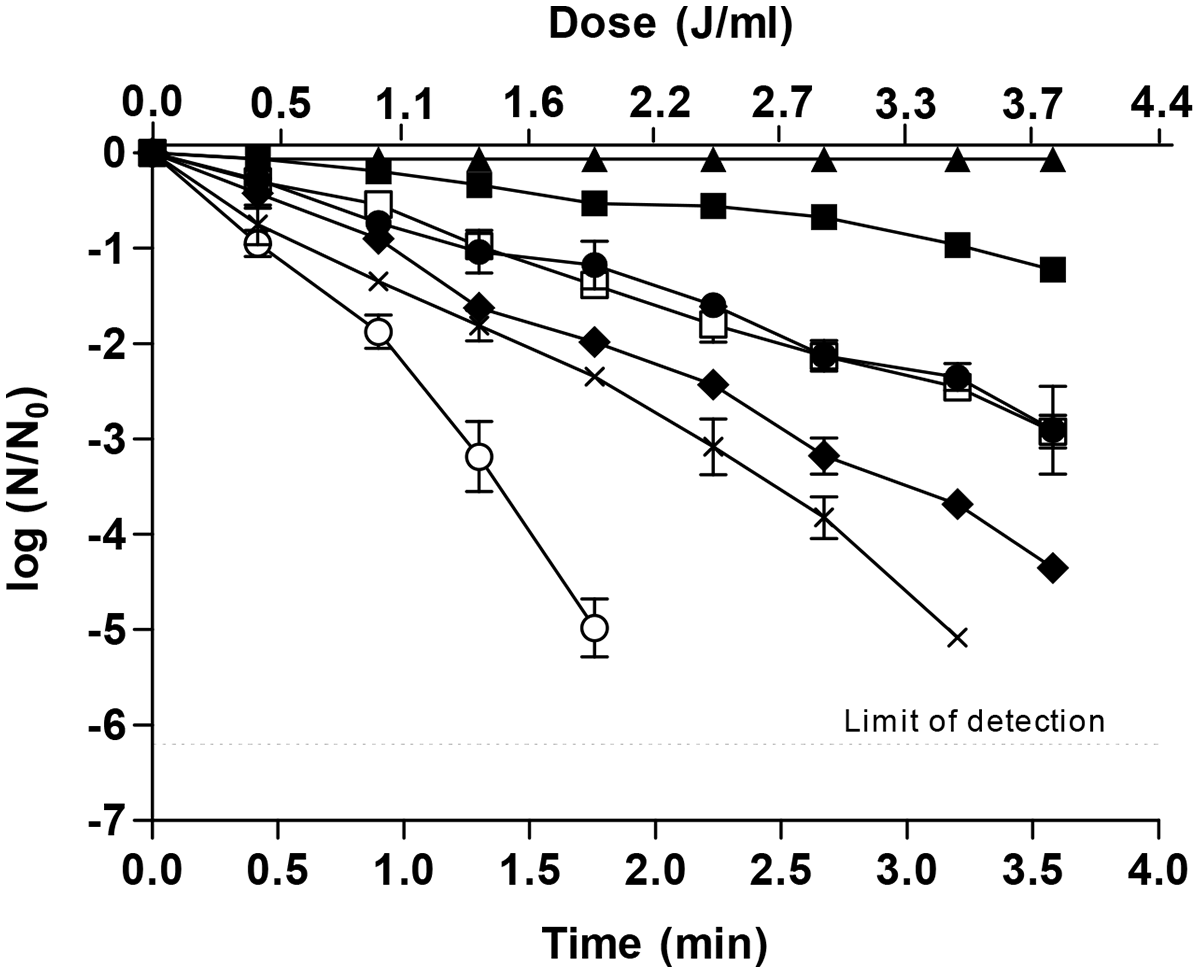
**Survival curves of *E. coli* STCC 4201 treated by DMDC (75 mg/L;▲), UV (■), heat (55°C;●), 55°C + 75 mg/L (♦), UV + 75 mg/L (☐), UV + 55°C (⨯), UV + 55°C 75 mg/L (❍)**.

In order to optimize the treatment conditions (UV dose and treatment time) required to achieve 5-log_10_ reductions of the investigated microorganism in apple juice by the combined process at 55°C in the presence of 75 mg/L of DMDC, survival curves where obtained (**Figure 2**). As observed, microbial inactivation increased with the treatment time for heat, UV or the different combined processes, as well as with the dose for the UV-based treatments. When UV was applied, survival curves showed an initial lag phase followed by an exponential inactivation rate, as has been described in the literature (Sastry et al., 2000; Koutchma et al., 2009; Gouma et al., 2015). However, the length of this shoulder was reduced when UV light was combined with heat, and the decrease was more noticeable when DMDC was added to the apple juice. Shoulder length has been related to damage repair capacity (Quintero-Ramos et al., 2004; López-Malo and Palou, 2005; Gayán et al., 2011, 2012c). Therefore, shorter shoulders could be due to a reduction in cellular capacity to repair damage or to the appearance of additional lethal damage that arises from the interaction of lesions induced by the applied hurdles. Since DMDC has been described as a powerful microbial inhibitor by inactivating cellular enzymes, and in this investigation the lethality of 75 mg/L DMDC was demonstrated when prolonging treatment exposition times over 10 min (**Figure 1**), this compound could impair cellular lesions with shorter exposition times, improving the effectiveness of UV-C light, heat or the combined process UV-H and diminishing the length of the shoulders or even causing them to disappear.

Independently of the kinetics of inactivation, **Figure 2** shows that the microbial inactivation of the individual treatments reached, in the best case, 3 log_10_ cycles. This lethality was increased when combining hurdles achieving near 5 log_10_ reductions when heat was applied in the presence of 75 mg/L of DMDC after 3.6 min or 5 log_10_ reductions when UV-C light (3.4 J/mL) was applied at 55°C over 3.2 min. However, the simultaneous application of UV-C light and heat (55°C) in the presence of 75 mg/L of DMDC reduced the treatment time and UV dose by up to 1.8 min and 1.96 J/mL, respectively, for a 5-log_10_ reduction of *E. coli* STCC 4201 in apple juice. That is, the addition of DMDC to the apple juice prior to the UV-Heat combined treatment reduced the processing time and the UV-C dose of the latter by 44%. Although the presence of 75 mg/mL of DMDC noticeably reduced the UV-H processing conditions needed to achieve a 5-log_10_ cycles reduction of the population of a UV-resistant microorganism like *E. coli* STCC 4201, more research is considered necessary to define processing conditions (treatment time, temperature, UV dose) more suitable for use on an industrial scale and to evaluate the lethal effectiveness of the combined process in other microorganisms. Moreover, DMDC has been proved to be an antimicrobial compound with a great lethal effect on bacteria like *E. coli* when applied alone, and even more when it was combined with heat and/or UV-C light. Therefore, its mechanism of inactivation in bacteria deserves further research when applied alone or in combined processes—like the one proposed in this investigation—in order to extend the application of such combined processes to other food products, enabling the inactivation of different kinds of microorganisms including yeast and Gram-positive and Gram-negative bacteria.

## Conflict of Interest Statement

The authors declare that the research was conducted in the absence of any commercial or financial relationships that could be construed as a potential conflict of interest.
